# Burden of Disease; the Current Status of the Diagnosis and Management of Atopic Dermatitis in China

**DOI:** 10.3390/jcm12165370

**Published:** 2023-08-18

**Authors:** Chaoying Gu, Xu Yao, Wei Li

**Affiliations:** 1 Department of Dermatology, Shanghai Institute of Dermatology, Huashan Hospital, Fudan University, Shanghai 200040, China; 2 Department of Allergy and Rheumatology, Jiangsu Key Laboratory of Molecular Biology for Skin Diseases and STIs, Hospital for Skin Diseases, Institute of Dermatology, Chinese Academy of Medical Sciences and Peking Union Medical College, Nanjing 210042, China

**Keywords:** atopic dermatitis, burden, prevalence, diagnosis, management

## Abstract

Atopic dermatitis (AD) is now a global health problem and has been attracting extensive attention from both academic and public society in China. This review aimed to present the current status of the prevalence, disease burden, clinical features, diagnosis, and management of AD in China. The prevalence of AD has been increasing rapidly in China during the past decades, partially due to the increased recognition of the disease; there are still substantial amounts of over-diagnosed eczema and under-diagnosed AD. Chinese dermatologists see many AD patients with atypical manifestation, which poses a challenge to the diagnosis. The Chinese diagnostic criteria for adults and pediatric patients with AD have been proposed respectively and validated with high sensitivity and specificity. International and Chinese guidelines for management of AD have been popularized; however, there are still many practices that need verification through randomized case-control study. Dupilumab and JAK inhibitors have demonstrated favorable efficacy for AD patients in China, and a multidimensional approach is needed for selection of the patients and evaluation of the efficacy and safety. Patient education and long-term management for AD are just beginning in China, and need to be strengthened in the future.

## 1. Introduction

Atopic dermatitis (AD), also known as atopic eczema, is a common chronic inflammatory skin disorder, with an increasing global incidence in the past few decades [1,2]. The pathophysiology of AD is complex and multifactorial, involving genetic disorders, skin barrier dysfunction, aberrant immune responses, and skin microbial dysbiosis. AD often develops during early infancy and childhood, and is characterized by eczematous lesions on the flexural areas, nape of the neck, and dorsum of the feet and hands. In adult patients, lichenified/exudative flexural dermatitis is common alone or with head, neck, and hand eczema, and (or) prurigo nodularis [3,4]. Patients with AD often experience itching, sleep disturbance, and abnormality in social, mental, and emotional function [5]. In particular, the chronic relapsing course of AD poses a significant negative effect on the quality of life of the patients and (or) their family caregivers [6,7,8]. AD is often concomitant with other atopic diseases, such as asthma, allergic rhinitis, and food allergies [9,10]. It can also increase the risk of other non-allergic comorbidities, such as many autoimmune- or immune-mediated diseases and mental disorders [11]. AD has become a global health issue since it causes high health-care costs worldwide and is associated with considerable morbidity and impairment of quality of life [12].

The prevalence of AD may vary by race: in the United States, the prevalence of AD in children is lower among whites (11%) than among African Americans (17%) [13,14]; the prevalence and persistence of AD in certain non-white racial/ethnic subgroups are higher than in non-Hispanic whites [13]. Studies have suggested that the incidence of AD is associated with industrialization. Over the past few decades, the overall prevalence of AD has increased two- to three-fold in industrialized countries, especially in Europe, the United States, and Japan, with the highest prevalence approaching 30% in some populations [15,16,17].

In China, dermatologists have made continuous efforts on research of AD in a Chinese context, from adapting the definition of AD to include eczema [18,19] to engaging in both clinical and basic research to cover the topics of AD relating to genetics and epigenetics [20,21], clinical and molecular phenotypes [22], immune response [23,24,25], the environment [26], and microbiomes [27,28,29]. This research has expanded our knowledge of the epidemiology, pathogenesis, clinical features, mechanism, interventions, and prognosis of the disease. As the concept of AD just began to popularize in China at the beginning of this century, the majority of Chinese dermatologists did not know the diagnosis and treatment of AD well. AD specialists in China have been engaging in popularizing the diagnosis and standard treatment of AD during the past two decades, which has resulted in substantial improvements. By consulting international guidelines/consensuses and conforming to Chinese situations, many guidelines and consensuses for AD diagnosis and management have been published; e.g., the Guideline for Diagnosis and Treatment of Atopic Dermatitis in China [30]. Continuing education based on these guidelines have greatly improved dermatologists’ knowledge on AD. Recently, with the progress in the treatment of AD by dupilumab and Janus kinase (JAK) inhibitors, more attention has been paid to AD. In this review, we aimed to report the current status of the prevalence, disease burden, clinical features, diagnosis, and management of AD in China.

## 2. Material and Methods

The study was performed following PRISMA guidelines for systematic reviews [31]. Related studies were searched from PubMed, Embase, and China National Knowledge Infrastructure (CNKI) until 31 December 2022. The search terms included “atopic dermatitis” OR “atopic eczema” OR “eczema” AND “Prevalence” OR “clinical features” OR “treatment” OR “therapies” OR “diagnosis”. Both English-language and Chinese-language publications were selected.

## 3. Result and Discussion

### 3.1. Prevalence of AD in China

Among children, the prevalence of AD ranges from 10% to 20% in developed countries [32]. During the past two decades, the prevalence of AD in China has been increasing greatly, approaching those of Western countries and developed Asian countries [33,34,35], although the increase is later than those of Japan [36] and Korea [37]. The prevalence of AD diagnosed using the United Kingdom (UK) working party criteria was 0.69% in adolescents (aged 6–20 years) in China in 1994 [38]. One study in Shanghai (China) district reported an prevalence of 8.3% in children aged 3–6 years in 2012 [26]. A study including twelve metropolises in China showed a prevalence of 12.94% in children aged 1–7 years in 2014, and the proportion of mild AD, moderate AD, and severe AD was 74.60%, 23.96%, and 1.44%, respectively. A more recent study reported that the prevalence of AD in infancy was 30.48% according to clinical diagnoses of dermatologists in China [19]. According to epidemiology data, the prevalence of AD in China is decreasing with age, which is also lower in rural areas. Passive smoking, premature birth, and choosy in food are risk factors for AD [33]. From 1990 to 2019, the number of patients with AD was increased by 25.65%, 3.85% of which was due to population aging, 20.16% due to population growth, and 1.64% due to age-specific prevalence [39]. The number of patients with AD was about 35.58 million in China in 2019 [39]. Childhood lifetime-ever eczema prevalence ranged from 10.0% to 30.0%; in total, 15.5 million children aged 3 to 6 years old and 70 million adolescent/adults aged 15 to 86 years old had lifetime-ever eczema in mainland China [40]. The prevalence of AD in China might be lower than that of Caucasians. A systematic review by Bylund et al. reported that the 1-year prevalence of doctor-diagnosed AD ranged from 1.2% in Asia to 17.1% in Europe in adults [41]. In a worldwide comparison, Swedish children showed the highest level of AD incidence (34%), and the lowest level was observed in Tunisian children (0.65%) [42].

### 3.2. Disease Burden of AD in China

As shown by the Global Burden of Disease Study, skin diseases are among the leading causes of the global burden of non-fatal diseases [43]. Compared to the general population, patients with AD report impaired health-related quality of life, accompanied by increased levels of itching, pain, sleep disturbances, anxiety, depression, and lower work productivity [44,45]. In younger patients, AD also has negative effects on the quality of life of their parents. Descriptions of the current status of disease burden of AD and its future trend would help to determine the direction and focus of public health, and provide policy-makers with data-based information to allocate health care resources accurately and effectively [46].

AD is a lifelong disease with a recurrence rate up to 75.9% within 7 years [47], placing huge and persistent economic and mental burdens on the patients and their families, and AD ranks first in the burden of non-fatal skin diseases [46]. Years lived with disability (YLD) reflects not only the incidence of the disease, but also the degree of disability caused by the disease. As a result, it gives a better indication of the severity of the epidemic. AD is a chronic disease with a big economic impact and affects both children and adults, potentially impairing productivity. In the case of non-fatal diseases, YLD is more suitable for estimating the burden of AD [48,49]. According to the Global Burden of Disease Study 2019 (GBD 2019), there were 171.17 million individuals and 7.48 million YLDs due to AD around the world in 2019. Among a total of 369 diseases, the percentage of YLDs due to AD was 0.87%, ranking twenty-eighth [39]. In China, among the 369 diseases, the age-standardized YLD rate of AD ranked twenty-fourth. From 1990 to 2019, the number of AD patients and YLD in China increased rapidly (1.43%) with population growth and aging.

The burden of disease can also be expressed in terms of disability-adjusted life years (DALYs), a measure of the difference between living in perfect health and living with an illness. DALYs are calculated as years lost due to disability or its consequences, plus the years of life lost due to premature mortality [50]. In non-fatal diseases such as AD, DALYs are measured primarily in the number of years lost to disability. The global DALY rate for AD was 121 in 1990 and 123 in 2017. China was among the five countries with the lowest age-standardized DALY rates (82.1, 44.2–138) due to AD in 2017 [46]. Generally, higher AD severity is associated with greater burdens on work productivity. Data reported by Andersen et al. show that patients with mild AD, moderate AD, and severe AD lose 2.4 h, 9.6 h, and 19.0 h per week, respectively [51]. Thus, AD improvement would reduce the economic burden.

### 3.3. Clinical Features of Chinese AD Patients

AD is a highly heterogeneous disease; infant, child, adolescent/adult, and elderly patients show significantly different manifestations, which are characterized by different preference in location and morphology of the skin lesions [52]. For example, adult AD patients often present with various manifestations, including flexural dermatitis, head-and-neck dermatitis, portrait dermatitis, hand dermatitis, nummular eczema, prurigo nodularis, generalized eczema, and erythroderma [8]. It is reported that different types of eczema manifestation showed differential responses to treatment. For example, dupilumab was effective for the treatment of nummular eczema-like AD, whereas patients with prurigo nodularis responded well to cyclosporine or dupilumab [8]. The presentation of AD may also vary among different ethnic origins [53]; however, systemic analyses for the differences in the clinical phenotypes of AD patients between Chinese and European-American populations are lacking. According to a recent study, the most commonly seen skin lesions of infantile AD in China are facial dermatitis (72.07%), xerosis (42.72%), and scalp dermatitis (27.93%) [19]. The features of elderly Chinese AD patients include widespread distribution of skin lesions in the trunk and extremities with “reversal sign” and less frequent concomitant atopic symptoms of AD [12]. The skin lesions of Asian (Japan and Korea) AD patients have higher proportions of T helper (Th) 17 inflammatory infiltration, in addition to Th2- and Th22-type inflammation, compared to the Th2/Th22-dominant inflammatory pattern in AD of the European-American population [54]. However, the molecular phenotype data of skin lesions for Chinese AD patients is still lacking. Nevertheless, it has been recently reported that Chinese moderate-to-severe AD patients are characterized by Th2-dominant serum biomarkers that are mixed with differentially increased Th1-, Th17-, and Th22-type cytokines/chemokines, and it is mainly Th2-type serum biomarkers that are positively correlated with disease severity and eosinophil counts. The patients can be grouped into two clusters that are differentially associated with inflammation [55]. Human filaggrin gene mutations are the most significant and well-replicated genetic mutation associated with AD. Wang et al. showed that among Chinese individuals, filaggrin P478S polymorphism might confer susceptibility to AD [56].

In China, patients with skin diseases usually go directly to see a dermatologist without recommendation by a family doctor, thus the manifestations of AD seen by Chinese dermatologists might be different from those in European or United States, which may lead to different understandings regarding AD. The typical manifestations of AD described in textbooks and by diagnosis criteria usually include infantile or child onset, dry skin, eczematous dermatitis at flexural sites—especially the antecubital fossa and popliteal fossa—and are usually accompanied with atopy such as food allergies, allergic rhinitis, and asthma. AD patients recommended by family doctors are usually severe and difficult to treat, and are mostly presented with typical manifestations. However, in China, the dermatologists see a lot of mild AD with manifestations that are not typical, and many of them are adult-onset or elderly-onset AD. A variety of inflammatory skin diseases are often accompanied by atopy and are actually AD; these include, but are not limited to, nummular eczema, prurigo nodularis, exfoliative cheilitis, eyelid dermatitis, hand and foot eczema, facial recurrent dermatitis, and seborrheic dermatitis. Most of these skin lesions are localized and the symptoms and signs are mild, but these manifestations do not prevent them from meeting the diagnostic criteria of AD. The skin lesions that are different from typical AD are called small signs or minor criteria of AD [57,58,59]; however, these patients still feature atopic signs and/or a personal history or family history of allergic disease, and many of them have elevated levels of serum total IgE or specific IgE. Atypical manifestations of AD differ from typical AD in terms of lesion type, distribution, and age of onset. In terms of morphology, atypical AD manifestations include nummular eczema, atopic prurigo, erythroderma, follicular AD, lichenoid AD, psoriasiform AD, and skin amyloidosis [60]. Atypical distribution indicates the extension sides of the trunk and extremities. As AD is common in children and was once considered a childhood disease that subsides after puberty [61], atypical AD by age includes adult-onset and elderly-onset AD. These atypical manifestations of AD pose a great challenge to diagnosis by Chinese dermatologists. In many cases, some of the patients with atypical manifestations are diagnosed with eczema, but not AD. The classical diagnosis criteria such as the Hanifin and Rajka criteria and UKWC are not sensitive in the diagnosis of AD in China; new diagnosis criteria for AD have been proposed and validated.

### 3.4. Diagnosis of AD in China

Currently, the diagnosis of AD is mainly based on symptoms, signs, and personal/family history of atopy, rather than definitive laboratory tests [62,63]. Several diagnostic criteria for AD have been proposed, including the Hanifin-Rajka (HR) criteria, the UK Working Party criteria, the modified American Academy of Dermatology (AAD) criteria, and the Japanese Dermatological Association (JDA) criteria [64,65]. Among various assessment methods of disease severity, SCORAD and EASI were widely used to evaluate the severity of the disease, and modified SCORAD and EASI were also developed for a more detailed understanding of the disease [66]. The differences in diagnosis criteria may explain the variability of the prevalence of AD reported in different studies. From December 2013 to February 2014, a study was conducted in twelve cities in China to explore the prevalence of AD [33]. According to the clinical diagnosis by experienced dermatologists participating in the study, the prevalence of AD was 12.94% nationwide. However, when the UK Working Party criteria and the HR criteria were used, the prevalence of AD in the same population was 4.76% and 3.51%, respectively [33]. A considerable number of mild cases did not meet the diagnostic criteria. Although the HR and UK Working Party criteria are the most widely used diagnostic criteria in clinical practice, their sensitivity and specificity are inconsistent in different populations [65,67,68,69], and the sensitivity is low in both adult and pediatric populations in China [18,19,70].

It is of note that these diagnostic criteria are not adequate for adult AD. The manifestation of adult AD is usually not as typical as that of children, and the diagnosis is often a challenge. In 2016, Dr. Jianzhong Zhang proposed the diagnostic criteria for adult AD in China: (1) eczema for more than 6 months; (2) personal and/or family history of atopic disease; (3) elevated total serum IgE level and/or positive allergen-specific IgE and/or eosinophilia. The first criterion plus either (2) or (3) can diagnose AD. The sensitivity of this criteria for diagnosis of AD in adolescents and adults is higher than that of the HR or UK Working Party criteria [18].

In infancy, AD presents with unique manifestations that are distinctive from children and adults. It is characterized by eczematous lesions on the cheeks, forehead or scalp, accompanied by intense itches leading to crusted erosions [71]. Because of this heterogeneity, diagnosis of infantile AD can be a challenge for dermatologists. The diagnostic criteria of AD widely used in epidemiological studies, e.g., HR and UK criteria, are not fully applicable in the infant group [71]. Guo et al. [19] established a novel diagnostic criteria of infants (children aged 1–12 months), which include: (1) onset after 2 weeks of birth; (2) pruritus and/or irritability and sleeplessness comparable with lesions; all two items above with one of the following items can reach a diagnosis of AD: (1) eczematous lesions distributed on cheeks and/or scalp and/or extensor limbs, and (2) eczematous lesions on any other parts of body accompanied by xerosis. This diagnostic criteria was shown to have a higher sensitivity and comparable specificity.

In 2020, Dr. Zhirong Yao proposed the Chinese diagnostic criteria for child AD: (1) pruritus; (2) typical morphology and site (flexor dermatitis) or atypical morphology and site accompanied by xeroderma; (3) chronic or chronic recurrent disease course. Typical morphology and sites (flexor dermatitis) include face and extremities of children. Atypical morphology and sites include: (1) typical eczematoid dermatitis occurring on the non-flexor side (scalp dermatitis, eyelid eczema, papilla eczema, nummular eczema, fingertip eczema, non-specific hand or foot dermatitis/atopic winter foot, nail or perinail eczema, and eczematoid rashes on other parts of the body); (2) atypical eczema rash (pityriasis simplex, cheilitis, subauricular and retroauricular/subnasal fissure, prurigo, pompholyx, papular lichenoid variation) [72]. Compared to classical HR and UK diagnostic criteria, the sensitivity of this diagnostic criteria was significantly higher in the epidemiological survey and the clinical setting, especially among mild and moderate AD. In birth cohorts, the new criteria showed similar sensitivity and specificity. In particular, the new diagnostic criteria for children also had a higher sensitivity in adult and elderly Chinese populations, especially for mild and moderate AD [73].

Although there have been great efforts in popularizing the new diagnosis criteria, the diagnosis rate of AD in China is still below what is anticipated. In China, a significant number of dermatologists believe that the diagnosis of AD must meet the criteria of the HR or UK Working Party criteria. AD patients that are mild, with atypical morphology and distribution of lesions, or with negative atopic history, are generally diagnosed with eczema [74]. Most dermatologists make the diagnosis of AD only when the clinical manifestations are typical, such as being symmetrical and having flexural dermatitis with dry skin accompanied by other allergic diseases. In particular, a positive family history of atopy is considered to be an important indicator for the diagnosis of AD [74]. A survey of 3016 Chinese dermatologists about the diagnosis for patients with symmetrical eczematous dermatitis reported that about half of the dermatologists claimed that more than 90% of these patients should be diagnosed with eczema and less than 10% were diagnosed as AD [18]. With the popularization of the new diagnosis criteria, the diagnosis rate of AD would increase in the near future.

### 3.5. Management of AD in China

**Figure 1:**
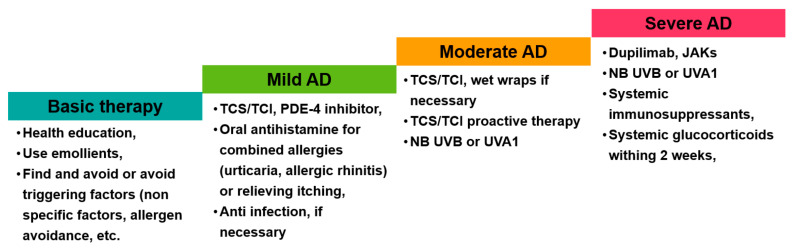
The flowchart summarizing treatment guidelines. JAK, Janus kinase; NB. Narrow band; PDE, phosphodiesterase; TCI, topical calcineurin inhibitor; TCS, topical corticosteoid; UV, ultraviolet rays.

The principle of AD treatment in China is severity-based stepped-care proposed by both international and Chinese guidelines for AD management. Basic treatments include application of emollients, avoidance of triggers (allergens and physical/chemical factors), and education of disease knowledge. The treatment guidelines were shown in Figure 1. The concept of emollient application has been wildly accepted by dermatologists in China, and more and more patients, especially pediatric patients with AD, use emollients regularly. However, there are still two wrong practices: one is inadequate use and the other is applying emollients on acute lesions. Emollients should be used adequately on chronic dry lesion or non-lesional skin. Patients with skin diseases including AD in China have a tradition of dietetic restraint, which mainly originates from the practice of traditional Chinese medicine. It is widely accepted by most AD patients and many dermatologists that certain foods, e.g., sea food, beef, mutton, and coriander, could induce or exacerbate the flares of AD. However, there is no evidence supporting the causal effects of stimulating food, and AD specialists in China have been struggling to educate patients and dermatologists against dietetic restraint. Food allergies are another concern of AD patients, which is indicated by allergen-specific IgE test. However, only food provocation tests can confirm the diagnosis of food allergy, and the actual food-induced flares of AD are not common, especially for adults [75]. The IgG test for food components is also used in China, but lacks scientific evidence. Most AD patients in China have been prescribed tests for total and (or) allergen-specific IgE. More than half of patients have elevated levels of total and (or) specific IgE (mostly dust mites) [18,76], though it is difficult to explain the results to the patients. The sensitization and skin inflammation of AD might be two parallel results of a common reason, and only in rare cases does the sensitization induce or exacerbate skin inflammation.

For mild-to-moderate AD, topical corticosteroid (TCS), topical calcineurin inhibitors (TCI), anti-histamines, and traditional Chinese medicine (TCM) are generally used in China, which are also recommended by the Chinese guidelines for AD management. TCS is recommended for control of the acute phase of skin lesion and the severe cases, and TCI is recommended for the mild and chronic phase, especially in the face, neck, and other sensitive body areas. There are two common wrong practices for the use of TCS: one is excessive use, which often results in side effects such as atrophy, striae, rosacea, telangiectasias, and purpura, and another is steroid phobia, which restricts the use of TCS and results in insufficient therapy. Proactive application with TCI or TCS is recommended for long-term maintenance, but it is difficult for patients to comply with this principle, and more efforts are needed to educate the patients. Chinese dermatologists have used a lot of TCI to treat AD in sensitive skin areas, whereas burning sensations, smaller packages, and relative high prices prevent the use of TCI. The topical phosphodiesterase 4 (PDE-4) inhibitor (crisaborole) has also been approved for AD treatment in China and demonstrates good efficacy for some patients, which has the advantages of favorable safety and moisturizing effects.

Anti-histamines are wildly used in China for itch relief and control of inflammation, as it is reported that anti-histamines also have anti-inflammatory effects [77]. While the effects of anti-histamines for AD are under debate in Europe and the United States [78], guidelines of AD management from Asian countries, including Japan and China, all recommend the use of anti-histamines [30,34]. More randomized case-control clinical studies are needed to justify the use of anti-histamines for AD. TCM is also widely used for AD treatment in China, which could improve the treatment of the disease and prevent recurrence if the patients are properly treated based on syndrome differentiation. The efficacies of several kinds of TCM have been tested in China, such as Xiao Feng San granules, Sheehan’s formula, and Jian Pi Shen Shi granules, which have all shown favorable efficacy in the treatment of AD [79]. For topical usage, several plant-derived compounds have been used in emollients, which have shown favorable effects on skin barrier dysfunctions and have been widely used by AD patients in China. However, the mechanisms of TCM in the treatment of AD remain unclear [80]. In reality, TCMs are currently overused in China, and the efficacy of many Chinese medicines have not been proved by clinical trials. Moreover, safety is also a concern. More randomized case-control clinical studies are needed in the future to verify the efficacy and safety of TCM for AD. Narrow-band ultraviolet B (NB-UVB) or ultraviolet A1 (UVA1) therapy is recommended for AD patients with chronic generalized lesions [30]. However, the use of UV therapy in China is limited, and should be popularized in the future, as this therapy is reliable and safe.

For moderate-to-severe AD patients, systemic treatments are required, which include corticosteroid; traditional immunosuppressants such as cyclosporine, methotrexate, azathioprine, and mortecophenolate; and newly introduced targeting therapies such as biologics and JAK inhibitors. Systemic corticosteroids are recommended for short-term use to control acute severe intractable lesions; however, long-term use is sporadically found being prescribed by primary doctors in China, and should be prohibited in the future. Many dermatologists in China in primary hospitals had limited experience in treating severe cases with AD, and had little experience using traditional systemic immunosuppressants. Even dermatologists in secondary and tertiary hospitals are reluctant to use systemic immunosuppressants because of the possible side effects. The most-used systemic drugs for AD in China are methotrexate and cyclosporine, and most of the patients receiving these medicines yield good outcomes and acceptable safety if monitored properly. With the approval of dupilumab, JAK inhibitors, upadacitinib, and abrocitinib for the treatment of AD in China, more dermatologists in primary hospitals can treat severe cases, as the new drugs are easy to use and safe. Dupilumab is effective in improving the signs and symptoms of moderate-to-severe Chinese AD patients [81]. We showed that dupilumab had favorable efficacy and well-tolerated safety in Chinese AD patients in real-world practice, with predicted good efficacy in females and predicted decreased responses in obese and elderly individuals to dupilumab [82]. We also revealed that high levels of serum CD25/sIL-2Rα, IL-31, and IL-36β might predict good efficacy of dupilumab treatment [56]. As IL-31 is a mediator of itches in AD, it is worth exploring the relationship between itches and anti-inflammation therapies for AD [83]. Long-term maintenance of AD using decreased doses of dupilumab may be the major practice in the future. Upadacitinib and abrocitinib are reported to be highly effective for AD, which are also suitable for moderate-to-severe AD patients who are not adequately controlled by other systemic drug products, including biologics [84]. As there are concerns over the safety of the JAK inhibitor, a comprehensive baseline screening should be performed before and during the treatment. However, dermatologists may have difficulty selecting these new systemic therapies. In clinical practice, efficacy, safety, and cost should be considered, and a decision would be made after adequate communication between the doctors and patients. Nevertheless, based on the good efficacy of dupilumab and JAK inhibitors, the treatment-to-target regimen has become realistic and feasible, and should be carried out in the future [85]. Allergic comorbidities of AD patients should be taken into account when selecting systemic treatment. It has been reported that dupilumab showed good efficacy for both the skin lesions and rhinosinusitis disease in AD patients accompanied with chronic rhinosinusitis, which would increase the compliance and benefit of the patients [86].

Patient education and long-term management are very important for AD patients, and are just in the beginning stages in China; there is a lot to do in the future. Currently, the aim of treatment focuses mainly on disease control in the acute stage, and less attention is paid to the maintenance treatment. Dermatologists are short on time and energy for long-term follow-up and management of patients. Regarding patients, there is insufficient disease cognition, poor treatment compliance, and low follow-up visit rates. Furthermore, there are few education programs for AD patients in China, and the patients lack an overall understanding of the etiology, manifestation, and treatment of AD. Therefore, patient education and long-term management should be strengthened in the future. The Chinese Society of Dermatologists have just published the consensus on whole-course management of atopic dermatitis, which would put forward the management of AD in China.

## 4. Strengths and Limitations

In the present study, we systematically reviewed the disease burden of AD in China with respect to disease prevalence, YLD, and DALYs, and also delineated the current status of the diagnosis and management of AD. However, studies about AD in China are still limited. Meanwhile, the studies in the review varied in terms of diagnostic criteria, experience of investigators, and heterogeneity of AD patients. Thus, the conclusions need to be verified by further studies.

## 5. Conclusions

AD is an important public health problem in China with high prevalence especially for children, causing serious social economic burdens. Despite recent advances in understanding the pathogenesis of AD, there are still many unmet needs in AD in China, including standard disease classification and accurate treatment strategy according to the classification of the disease. Thus, more accurate diagnosis of AD is needed, irrespective of the atypical manifestation. Evidence-based management is recommended to reduce the subjectivity in the treatment. More emphasis needs to be placed on patient education and long-term management.

## Data Availability

Not applicable.
